# Chronic Central Adrenal Insufficiency in a Patient With Remote Traumatic Brain Injury and an Apparently Adequate Standard‐Dose ACTH Response: A Case Report

**DOI:** 10.1002/ccr3.73582

**Published:** 2026-09-24

**Authors:** Kazunori Kageyama, Keisuke Sato, Satoshi Yamagata, Mizuki Tasso, Yuki Nakada, Shojiro Sawada

**Affiliations:** ^1^ Division of Diabetes, Metabolism and Endocrinology, Faculty of Medicine Tohoku Medical and Pharmaceutical University Sendai Miyagi Japan

**Keywords:** ACTH stimulation test, central adrenal insufficiency, hypopituitarism, traumatic brain injury

## Abstract

In patients with a remote history of traumatic brain injury, central adrenal insufficiency may remain unrecognized for decades. An apparently adequate response to standard‐dose ACTH stimulation should not preclude further evaluation of the hypothalamic–pituitary–adrenal axis when clinical and biochemical findings continue to suggest adrenal insufficiency.

## 1 Introduction

Hypopituitarism can result from various conditions, including pituitary tumors, surgery, radiation therapy, infiltrative diseases, infections, vascular disorders, and genetic defects [1]. Traumatic brain injury (TBI) is also a well‐recognized cause of hypopituitarism [2] but may be overlooked, particularly when endocrine manifestations develop long after the initial injury. TBI can damage the hypothalamic–pituitary system directly through mechanical shearing forces or indirectly through vascular injury resulting in ischemia or infarction [3, 4]. Post‐traumatic hypopituitarism may develop during the acute phase or become clinically apparent months to years after the injury [5, 6, 7].

Coronavirus disease 2019 (COVID‐19) has also been associated with hypothalamic–pituitary dysfunction [8]. Cases of new‐onset central adrenal insufficiency following COVID‐19 have been reported [9, 10]. However, in patients with preexisting risk factors for pituitary dysfunction, systemic illness, such as COVID‐19, may also reveal previously unrecognized endocrine abnormalities rather than directly causing them.

Herein, we report a patient with a remote history of severe TBI in whom central adrenal insufficiency became clinically apparent following COVID‐19. Because longitudinal endocrine data were unavailable, the relative contributions of remote TBI and COVID‐19 could not be established. Despite an apparently adequate cortisol response to a standard‐dose ACTH stimulation test, subsequent evaluation demonstrated impaired hypothalamic–pituitary–adrenal (HPA) axis function. This case highlights that, in patients with a remote history of TBI, central adrenal insufficiency may remain unrecognized for years and that a standard‐dose ACTH stimulation test may not exclude subtle or chronic impairment of the HPA axis.

## 2 Case History/Examination

A 54‐year‐old man was referred to our hospital because of severe fatigue and hyponatremia. One year before the current presentation, he had been admitted to our department with hyponatremia that developed 7 days after COVID‐19 infection. Adrenal insufficiency was suspected, and hydrocortisone replacement therapy was initiated. A standard‐dose ACTH stimulation test using synthetic ACTH (tetracosactide, 250 μg) was performed. Serum cortisol concentrations were measured by electrochemiluminescence immunoassay (ECLIA) at SRL Inc. (Tokyo, Japan). Serum cortisol concentrations were 6.72 μg/dL at baseline, 15.0 μg/dL at 30 min, and 18.0 μg/dL at 60 min. At that time, a peak cortisol concentration of ≥ 18.0 μg/dL was used at our institution as the conventional criterion for an adequate response; hydrocortisone was therefore discontinued.

One year later, the patient developed severe fatigue and again sought medical attention. Marked hyponatremia (serum sodium, 118 mEq/L) was detected, and he was referred to our department. His Glasgow Coma Scale score was 15. His medical history was notable for severe TBI sustained in a motorcycle accident at 20 years of age, more than 30 years before the current presentation. He had lost consciousness for 2 days and had also sustained fractures of the clavicle, ribs, and sternum.

On admission, his height was 165.1 cm, his body weight was 58.0 kg, and his body mass index was 21.3 kg/m^2^. His blood pressure was 146/79 mmHg, heart rate was 61 beats/min, and body temperature was 35.9°C. Laboratory examination revealed hyponatremia and hypochloremia (serum sodium, 132 mEq/L; chloride, 96 mEq/L) and mild anemia (hemoglobin, 13.2 g/dL) (Table 1). There were no physical findings suggestive of heart failure, renal failure, or nephrotic syndrome.

**TABLE 1:** Laboratory data on admission.

	On admission	(Reference range)
Peripheral blood		
White blood cells (/μL)	3400	(3300–8600)
Red blood cells (/μL)	415 × 10^4^	(435–555 × 10^4^)
Hemoglobin (g/dL)	13.2	(13.7–16.8)
Hematocrit (%)	38.6	(40.7–50.1)
Platelets (/L)	182 × 10^3^	(158–348 × 10^3^)
Blood biochemistry		
Na (mEq/L)	132	(138–145)
K (mEq/L)	4.4	(3.6–4.8)
Cl (mEq/L)	96	(101–108)
Osmolality (mOsm/kg H_2_O)	268	(257–290)
Uric acid (mg/dL)	3.5	(3.7–7.8)
Glucose (mg/dL)	99	(73–109)
BUN (mg/dL)	7	(8–20)
Creatinine (mg/dL)	0.65	(0.65–1.07)
Glucose (mg/dL)	99	(70–109)
HbA1c (%)	5.4	(4.9–6.0)
Albumin (g/dL)	4.9	(4.1–5.1)
AST (IU/L)	30	(13–30)
ALT (IU/L)	23	(10–42)
γ‐GTP (IU/L)	28	(10–47)
Alkaline phosphatase (IU/L)	55	(38–113)
LDL cholesterol (mg/dL)	159	(65–163)
HDL cholesterol (mg/dL)	49	(38–90)
Triglyceride (mg/dL)	105	(40–234)
Urinary biochemistry		
Na (mEq/L)	242	—
K (mEq/L)	38.6	—
Cl (mEq/L)	232	—
Osmolality (mOsm/kg H_2_O)	688	(50–1300)
Hormonal data		
TSH (μU/mL)	6.05	(0.61–4.23)
Free T_3_ (pg/mL)	2.09	(2.30–4.00)
Free T_4_ (ng/dL)	0.71	(0.90–1.70)
ACTH (pg/mL)	28.7	(7.2–63.3)
Cortisol (μg/dL)	6.62	(7.07–19.60)
DHEA‐S (μg/dL)	43	(76–386)
AVP (pg/mL)	1.1	(< 2.8)
PAC (pg/mL)	29.7	(4.0–82.1)
PRA (ng/ml/h)	1.2	(0.2–2.3)
LH (mIU/mL)	1.23	(0.79–5.72)
FSH (mIU/mL)	2.75	(3.00–14.70)
Testosterone (ng/mL)	3.51	(2.00–8.30)
PRL (ng/mL)	4.86	(3.58–12.78)
GH (ng/mL)	< 0.03	(0.10–2.47)
IGF‐1 (ng/mL)	45	(84–239)
Urinary free cortisol (μg/day)	< 0.1	(4.3–176)
TgAb	1450	(0–19)
TPOAb	86.6	(0–3)

*Note:* Reference ranges are not established for spot urinary electrolyte concentrations.

Abbreviations: γ‐GTP, gamma‐glutamyl transpeptidase; ACTH, adrenocorticotropic hormone; ALT, alanine aminotransferase; AST, aspartate aminotransferase; AVP, arginine vasopressin; BUN, blood urea nitrogen; DHEA‐S, dehydroepiandrosterone sulfate; FSH, follicle‐stimulating hormone; GH, growth hormone; HbA1c, hemoglobin A1c; HDL, high‐density lipoprotein; IGF‐1, insulin‐like growth factor‐1; LDL, low‐density lipoprotein; LH, luteinizing hormone; PAC, plasma aldosterone concentration; PRA, plasma renin activity; PRL, prolactin; T_3_, triiodothyronine; T_4_, thyroxine; TgAb, antithyroglobulin antiboby; TPOAb, antithyroid peroxidase antibody; TSH, thyroid‐stimulating hormone.

## 3 Methods

### 3.1 Differential Diagnosis and Investigations

**FIGURE 1:**
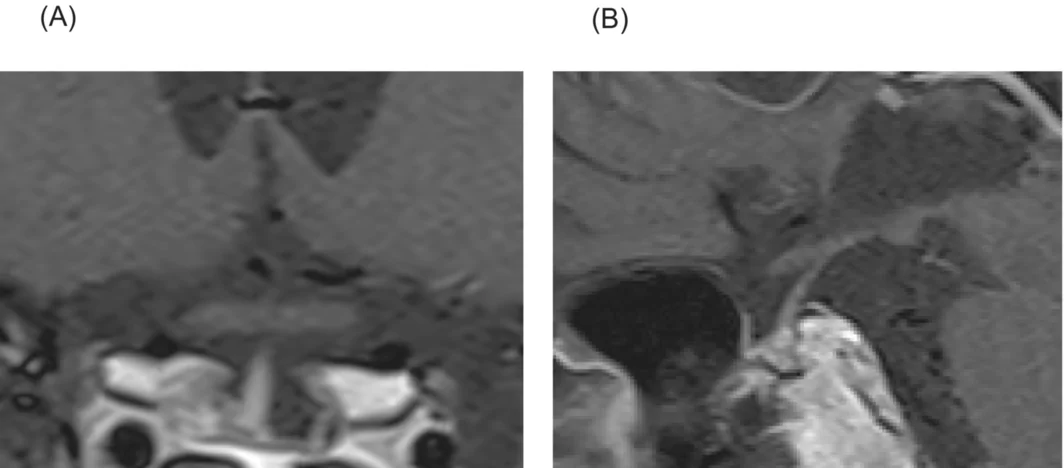
Pituitary magnetic resonance imaging (MRI). (A) Coronal section. (B) Sagittal section. Pituitary gadolinium‐enhanced T_1_‐weighted MRI demonstrating an empty sella. The pituitary stalk is deviated to the right. A deformation of the posterior clinoid process is identified.

Pituitary magnetic resonance imaging revealed an empty sella with rightward deviation of the pituitary stalk and deformation of the posterior clinoid process (Figure 1). Abdominal computed tomography demonstrated bilateral adrenal atrophy (data not shown). Serum sodium increased to 136 mEq/L on the day after admission, allowing further endocrinological evaluation.

Basal endocrine assessment suggested hypothalamic–pituitary dysfunction (Table 1). The patient had hypotonic hyponatremia (serum sodium, 132 mEq/L; serum osmolality, 268 mOsm/kg H_2_O) with concentrated urine (urine osmolality, 688 mOsm/kg H_2_O) and a high urine sodium concentration (242 mEq/L). Serum uric acid (3.5 mg/dL) and blood urea nitrogen (7 mg/dL) were also low. The plasma arginine vasopressin (AVP) concentration was not suppressed (1.1 pg/mL) despite hypo‐osmolality, indicating an AVP‐mediated impairment of free‐water excretion. Although these findings were biochemically compatible with an SIADH‐like pattern, primary SIADH was not diagnosed because cortisol deficiency can itself impair AVP suppression and produce similar hypotonic hyponatremia. There were no clinical findings suggestive of heart failure, renal failure, or nephrotic syndrome. Primary hypothyroidism was also present and may have contributed to the hyponatremia; however, the markedly reduced cortisol production supported central adrenal insufficiency as an important contributor. The 24‐h urinary free cortisol (UFC) level was undetectable (< 0.1 μg/day). Thyroid hormone levels were decreased with an elevated thyroid‐stimulating hormone (TSH) concentration, and both antithyroglobulin and antithyroid peroxidase antibodies were strongly positive, supporting autoimmune primary hypothyroidism rather than central hypothyroidism (Table 1).

### 3.2 Dynamic Endocrine Testing

To further evaluate pituitary function, combined corticotropin‐releasing hormone (CRH), luteinizing hormone‐releasing hormone, and thyrotropin‐releasing hormone stimulation tests were performed. CRH stimulation elicited a preserved but delayed ACTH response accompanied by an insufficient cortisol response, with a peak cortisol concentration of 11.6 μg/dL (Table 2), a pattern described in hypothalamic hypopituitarism [11]. Because CRH testing alone cannot definitively localize the lesion, this finding was interpreted as compatible with hypothalamic or upper pituitary stalk dysfunction rather than as definitive localization. TSH showed a marked response to TRH (6.24–40.4 μU/mL), consistent with preserved thyrotroph reserve in the setting of primary hypothyroidism, whereas the responses of the other evaluated pituitary hormones were preserved. A growth hormone (GH)‐releasing peptide‐2 (GHRP‐2) stimulation test showed a peak GH concentration of 6.11 ng/mL (Table 2). Chihara et al. validated a peak GH cutoff of 9 ng/mL during GHRP‐2 stimulation for severe adult GH deficiency [12]; therefore, the present peak GH concentration met this biochemical criterion.

**TABLE 2:** Endocrinological stimulation tests during the previous and current admissions.

CRH + LHRH + TRH test				
Time (min)	0	30	60	90
ACTH (pg/mL)	27.2	53.5	57.4	35.1
Cortisol (μg/dL)	5.7	9.7	11.6	10.5
LH (mIU/mL)	1.72	14.7	13.1	10.5
FSH (mIU/mL)	3.50	5.79	6.07	6.05
TSH (μU/mL)	6.24	40.4	28.1	20.6
PRL (ng/mL)	5.88	11.4	5.48	5.14

GHRP‐2 test					
Time (min)	0	15	30	45	60
GH (ng/mL)	0.13	5.75	6.11	3.57	1.60

Previous standard‐dose ACTH stimulation test (1 year earlier)	(250 μg)		
Time (min)	0	30	60
Cortisol (μg/dL)	6.72	15.0	18.0

Low‐dose ACTH stimulation test (current admission)	(1 μg)		
Time (min)	0	30	60
Cortisol (μg/dL)	3.7	11.2	10.9

*Note:* During the current admission, CRH, LHRH, TRH, GHRP‐2, and low‐dose synthetic ACTH (tetracosactide) were administered intravenously at doses of 100, 100, 500, 100, and 1 μg, respectively. The previous standard‐dose ACTH stimulation test used 250 μg tetracosactide. For the low‐dose ACTH test, 250 μg/2 mL tetracosactide was diluted with 23 mL of 0.9% saline (10 μg/mL); 1 mL was then further diluted with 9 mL of saline (1 μg/mL), and 1 mL (1 μg) was administered intravenously. Serum cortisol was measured at 0, 30, and 60 min.

Abbreviations: ACTH, adrenocorticotropic hormone; CRH, corticotropin‐releasing hormone; FSH, follicle‐stimulating hormone; GH, growth hormone; GHRP‐2, growth hormone‐releasing peptide‐2; LH, luteinizing hormone; LHRH, luteinizing hormone‐releasing hormone; PRL, prolactin; TRH, thyrotropin‐releasing hormone; TSH, thyroid‐stimulating hormone.

Given the discrepancy between the previous standard‐dose ACTH test and the subsequent findings suggestive of chronic HPA axis impairment, a low‐dose ACTH stimulation test was performed. After 30 min of rest, tetracosactide (250 μg/2 mL) was diluted with 23 mL of 0.9% saline to 10 μg/mL; 1 mL of this solution was further diluted with 9 mL of saline to 1 μg/mL. After baseline sampling, 1 mL (1 μg) was administered intravenously, and serum cortisol was measured at 0, 30, and 60 min. Cortisol concentrations were 3.7, 11.2, and 10.9 μg/dL, respectively (Table 2), indicating impaired adrenal responsiveness when interpreted together with the other findings. Taken together, the delayed ACTH response to CRH, inadequate cortisol responses to CRH and low‐dose ACTH stimulation, undetectable UFC, and bilateral adrenal atrophy supported chronic central adrenal insufficiency associated with hypothalamic–pituitary dysfunction. Severe GH deficiency was also diagnosed.

### 3.3 Treatment

Hydrocortisone replacement therapy was initiated at 15 mg/day. Nine days later, levothyroxine (25 μg/day) was added for primary hypothyroidism.

## 4 Conclusions and Results (Outcome and Follow‐Up)

The patient's clinical condition and serum sodium concentration improved after hormone replacement. After obtaining informed consent for GH replacement therapy, once‐weekly somapacitan was initiated at 1.5 mg approximately 2 months after discharge and subsequently increased to 2.0 mg.

## 5 Discussion

Herein, we describe chronic hypothalamic–pituitary dysfunction in a patient with a remote history of severe TBI whose central adrenal insufficiency became clinically apparent after COVID‐19. More than 30 years before the current presentation, the patient had sustained severe head trauma with loss of consciousness for 2 days. Endocrinological evaluation demonstrated a preserved but delayed ACTH response to CRH accompanied by an insufficient cortisol response, compatible with dysfunction at the hypothalamic or upper pituitary stalk level [11]. The undetectable UFC level and bilateral adrenal atrophy further supported prolonged impairment of the HPA axis rather than an acute process. In addition, the GHRP‐2 test demonstrated severe GH deficiency. Taken together, these findings supported chronic hypothalamic–pituitary dysfunction involving ACTH and GH secretion.

The recurrent hyponatremia required careful differential diagnosis. At the current admission, the combination of hypotonic hyponatremia, concentrated urine, high urine sodium, low uric acid, low blood urea nitrogen, and inadequately suppressed AVP was compatible with an SIADH‐like biochemical pattern. However, secondary adrenal insufficiency can cause hyponatremia through failure of cortisol‐mediated suppression of AVP and impaired free‐water excretion, thereby mimicking SIADH [13]. Primary hypothyroidism was also present, although clinically important hyponatremia is generally associated with more severe hypothyroidism [13]. Hypervolemic causes were considered less likely because there were no findings suggestive of heart failure, renal failure, or nephrotic syndrome. Thus, primary SIADH could not be established, and central adrenal insufficiency was considered an important contributor to the recurrent hyponatremia, with a possible additional contribution from primary hypothyroidism.

An important clinical feature of this case was the discrepancy between the standard‐dose and low‐dose ACTH stimulation tests. One year before the current evaluation, a standard‐dose (250 μg) ACTH stimulation test yielded a peak cortisol concentration of 18.0 μg/dL, which was considered an adequate response according to the conventional criterion used at our institution, and hydrocortisone was discontinued. Serum cortisol was measured by ECLIA. The historical stimulated cortisol cutoff of 18 μg/dL was established using older cortisol assays, whereas lower assay‐specific thresholds have been proposed for newer, more specific immunoassays [14]. Therefore, the previous value of 18.0 μg/dL should be regarded as an apparently adequate response according to the criterion used at that time rather than as universal evidence of normal HPA axis function. In addition, the standard‐dose ACTH test provides a supraphysiological stimulus that markedly exceeds normal circulating ACTH concentrations [15] and may preserve an apparently adequate cortisol response in patients with subtle or partial central adrenal insufficiency [16]. The low‐dose ACTH test has been proposed as a more sensitive approach in selected clinical settings [17]. In the present case, the subsequent low‐dose cortisol peak of 11.2 μg/dL, delayed ACTH response to CRH with an insufficient cortisol response, undetectable UFC, and bilateral adrenal atrophy provided converging evidence of chronic central adrenal insufficiency. Thus, when clinical findings remain suggestive despite an apparently adequate standard‐dose ACTH response, further assessment of the HPA axis should be considered rather than relying on a single stimulation test.

Post‐traumatic hypopituitarism is an important but often underrecognized consequence of TBI. Meta‐analyses have suggested that approximately 20%–30% of patients develop some degree of pituitary dysfunction following TBI [3, 18]. Although pituitary dysfunction frequently becomes evident within the first several months after injury, cases diagnosed more than a decade later have also been reported [19, 20]. Several mechanisms have been proposed, including direct mechanical damage to the hypothalamus, pituitary stalk, or pituitary gland; vascular injury resulting in ischemia or infarction; and post‐traumatic autoimmune mechanisms [3, 4, 19]. Antipituitary antibodies have been detected in a substantial proportion of patients after TBI, suggesting that autoimmune processes may contribute to persistent or progressive pituitary dysfunction in some patients [21]. In Japan, the interval between head trauma and the recognition of post‐traumatic hypopituitarism has been reported to range from the acute phase to as long as 31 years [20, 22, 23]. These observations indicate that a remote history of significant head trauma remains clinically relevant when evaluating otherwise unexplained pituitary hormone deficiencies, although they do not establish causality in an individual patient.

The structural abnormalities observed on pituitary magnetic resonance imaging may be compatible with previous injury to the hypothalamic–pituitary region. The patient had an empty sella, rightward deviation of the pituitary stalk, and deformation of the posterior clinoid process. However, these findings are nonspecific and cannot establish either the etiology or timing of pituitary dysfunction. There was also no evidence allowing us to establish a preexisting pituitary tumor or previous pituitary apoplexy. Accordingly, the imaging findings were considered supportive contextual findings rather than diagnostic evidence that the remote TBI caused the current hypothalamic–pituitary dysfunction.

This case has several limitations. Pituitary function was not evaluated before or immediately after the TBI, and no longitudinal endocrine data were available during the intervening decades; therefore, the onset and progression of pituitary dysfunction cannot be determined, and a causal relationship with the remote TBI cannot be established. Similarly, pituitary function immediately before COVID‐19 was unknown. COVID‐19 was temporally associated with the first recognized episode of severe hyponatremia and may have contributed to or unmasked previously unrecognized adrenal insufficiency. Therefore, the relative contributions of remote TBI and COVID‐19 remain uncertain. In addition, the diagnosis of chronic central adrenal insufficiency was based on the combined clinical, biochemical, dynamic testing, and imaging findings rather than a single definitive diagnostic test. Nevertheless, the concordance of the delayed ACTH response to CRH, inadequate cortisol responses, undetectable UFC, and bilateral adrenal atrophy strongly supported chronic impairment of the HPA axis.

In conclusion, this case illustrates that central adrenal insufficiency may remain unrecognized for years in patients with a remote history of TBI and may become clinically apparent during systemic illness. An apparently adequate cortisol response to a standard‐dose ACTH stimulation test does not necessarily exclude subtle or chronic central adrenal insufficiency when other clinical and biochemical findings remain suggestive. In such cases, comprehensive assessment of the HPA axis, including additional dynamic testing when appropriate, may facilitate timely diagnosis and treatment and help prevent potentially life‐threatening adrenal insufficiency.

## Author Contributions

**Kazunori Kageyama:** conceptualization, writing – original draft, writing – review and editing, formal analysis, project administration. **Keisuke Sato:** data curation, investigation, validation. **Satoshi Yamagata:** investigation, validation. **Mizuki Tasso:** data curation, investigation. **Yuki Nakada:** data curation, investigation, validation. **Shojiro Sawada:** supervision, writing – review and editing.

## Funding

The authors have nothing to report.

## Ethics Statement

This study was conducted in accordance with the principles of the Declaration of Helsinki. Ethical approval was not required for this single case report in accordance with institutional policy.

## Consent

Written informed consent was obtained from the patient for publication of this case report and any accompanying images.

## Conflicts of Interest

The authors declare no conflicts of interest.

## Data Availability

The data that support the findings of this study are available on request from the corresponding author. The data are not publicly available due to privacy or ethical restrictions.
